# Complete Genome Sequences of Streptomyces lividans Bacteriophages Satis and JustBecause

**DOI:** 10.1128/MRA.00501-19

**Published:** 2019-08-08

**Authors:** Kelly Hartigan, John Hudson Alarcon, Andrew Cao, Yongzhen Chen, Nicole Curnutt, Brooke Felsheim, Saransh Gothi, Lucia Grandison, Matthew McDermut, Kaeli-Skye Spight, Xuejing Xu, Kathleen Weston-Hafer, Christopher Shaffer

**Affiliations:** a Department of Biology, Washington University in St. Louis, St. Louis, Missouri, USA; Queens College

## Abstract

We present here the complete genomes of two *Streptomyces* bacteriophages, Satis and JustBecause. Both phages were isolated directly from soil samples collected in St. Louis, MO, and present with an unusual prolate head morphology and large genome lengths of over 180 kb.

## ANNOUNCEMENT

Given the immense diversity of mycobacteriophages in the biosphere, the sequencing and annotation of actinobacteriophage genomes continue to offer insight into viral evolution and genetic diversity within the population. Bacteriophages can be grouped into clusters based on overall nucleotide sequence similarity and shared gene content as identified by Phamerator using pairwise sequence alignment of coding genes (1, 2). These two phages, Satis and JustBecause, are the founding members of a cluster of genetically similar *Streptomyces* phages (the BM cluster in the Actinobacteriophage Database) (3) and represent a significant sequence diversion from previously published phages.

Satis and JustBecause were isolated using direct plating of aqueous supernatant from soil samples collected in St. Louis, MO, on cultured spores of Streptomyces lividans JI 1326. Isolation and growth conditions followed published protocols (https://phagesdb.org/workflow/) with the exception of the use of Streptomyces lividans as the phage host. DNA was purified using a Wizard genomic DNA purification kit, and the library was created using a KAPA Biosystems high-throughput library preparation kit. Both strains were multiplexed and sequenced using the Illumina MiSeq platform to produce a total of 87,633 and 77,507 paired-end 300-bp reads for Satis and JustBecause, respectively. Reads were analyzed using FastQC ver. 0.11.5, which reported no issues of concern. Reads were then trimmed with Trimmomatic ver. 0.36 using default settings (4), and paired-end reads were merged with PEAR ver. 0.9.10 (5) prior to assembly with Newbler ver. 2.7 using default settings. Assemblies were then visually assessed for quality with Consed ver. 27 (6). The resulting assemblies contained shotgun coverage that typically ranged from ∼100-fold to ∼200-fold (see https://openscholarship.wustl.edu/data/19/ and https://openscholarship.wustl.edu/data/18). Physical ends were determined based on a buildup of reads with identical ends (328 reads for Satis, 314 reads for JustBecause). The coverage between these two identified ends was ∼2-fold higher than the remainder of the assembly, strongly suggesting phage genomes with defined termini and terminal repeats of 1,049 and 1,043 bp for Satis and JustBecause, respectively (see https://openscholarship.wustl.edu/data/19/ and https://openscholarship.wustl.edu/data/18 for exact details). Based on these assemblies, Satis and JustBecause have exceptionally long genomes for bacteriophages, at 186,702 and 184,281 bp, respectively, with a 66.6% GC content. As confirmed through electron microscopy (EM) imaging, each phage belongs to the family *Siphoviridae*, but they have atypical large, prolate capsids (for EM images see http://phagesdb.org/phages/satis and http://phagesdb.org/phages/justbecause).

The coding genes for each genome were identified using predictions from GLIMMER (7) and GeneMark (8) followed by manual inspection and improvement as needed. Satis and JustBecause have 324 and 325 protein-coding genes and 13 and 14 tRNAs, respectively. These genes were functionally annotated using BLAST (9), HHpred (10), and Phyre2 (11). tRNAs were identified using tRNAscan-SE (12) and ARAGORN (13).

As the founding members of Cluster BM, Satis and JustBecause show striking differences from other sequenced phages, as well as differences from each other. Initial analysis of the Satis annotated proteins by Phamerator clustered only 25 of the 324 (7.72%) proteins in Satis to proteins previously seen in other actinobacteriophages (1). Furthermore, while Satis and JustBecause have been classified in the same cluster, the genomes of these two phages differ considerably from each other. They have a calculated average nucleotide identity (ANI) of only 74.4% (14). Morphologically, the large genomes of these two phages correlate with their unusually large capsids, with Satis having an average capsid length of 285 ± 4.7 nm (*n* = 5 ± 2 standard error of the mean [SEM]) and a width of 47 ± 1.9 nm (*n* = 5 ± 2 SEM). The annotation of these two novel genomes shows that the limit of genomic diversity has yet to be reached.

### Data availability.

The genome sequences of Satis and JustBecause are available in GenBank under accession numbers MH576962 and MH744418, respectively. The raw reads and full assemblies for Satis and JustBecause are also available at https://openscholarship.wustl.edu/data/19/ and https://openscholarship.wustl.edu/data/18, respectively.
